# A Comparison of Cognitive-Behavioral Therapy and Pharmacotherapy vs. Pharmacotherapy Alone in Adults With Attention-Deficit/Hyperactivity Disorder (ADHD)—A Randomized Controlled Trial

**DOI:** 10.3389/fpsyt.2018.00571

**Published:** 2018-11-16

**Authors:** Salvatore Corbisiero, Hannes Bitto, Patricia Newark, Beatrice Abt-Mörstedt, Marina Elsässer, Jacqueline Buchli-Kammermann, Sven Künne, Elisabeth Nyberg, Maria Hofecker-Fallahpour, Rolf-Dieter Stieglitz

**Affiliations:** ^1^Division of Clinical Psychology and Psychiatry, University of Basel Psychiatric Clinics, Basel, Switzerland; ^2^Division of Clinical Psychology and Psychiatry, Department of Psychology, University of Basel, Basel, Switzerland

**Keywords:** ADHD, cognitive-behavioral therapy, multimodal therapy, pharmacotherapy, standard clinical management, adulthood

## Abstract

In the treatment of adult attention-deficit/hyperactivity disorder (ADHD) the importance of psychological interventions in combination with pharmacotherapy is widely accepted in contemporary clinical routine. The natural course of the disorder seems to justify additional psychological interventions because even in patients who are highly compliant to pharmacotherapy full remission is not always achieved. The aim of the present study was to analyze the contribution of psychotherapy to the treatment of adult ADHD patients. In a randomized controlled study, the efficacy of a combined treatment of psychotherapy with pharmacotherapy is compared to pharmacological intervention alone. After initiation and stabilization of treatment with methylphenidate (MPH) in all subjects randomization to the two different treatment conditions was done. Afterwards both groups underwent treatment for about 10–12 weeks, the experimental group receiving sessions of cognitive-behavioral therapy (CBT) whereas the control group only received medication and standard clinical management (SCM). ADHD symptoms differed statistically during time but not between the two different treatment conditions. This result was the same for the single ADHD symptoms—inattention, hyperactivity, impulsivity, and emotional symptoms—and also for impairment. Individual standardized ADHD specific CBT program was not able to outperform SCM.

## Introduction

Attention-deficit/hyperactivity disorder (ADHD) beginning in childhood has been accepted to persist in ~50% of cases into adulthood (1). With a worldwide prevalence between 2.5 and 4.4% (2–4) ADHD is a frequent disorder in adulthood. The disorder is characterized by the core symptoms inattention, impulsivity, and hyperactivity (5–7), and additional symptoms such as emotional dysregulation [see (8)]. These symptoms have often a substantial impact on daily functioning leading to severe impairment in different domains of life (9, 10). According to guidelines (11–13) psychopharmacological treatment is the first line treatment in adult ADHD, but a multimodal approach is generally recommended, involving educational aspects about the disorder and psychotherapy addressing concomitant problems (12–14).

ADHD is a multi-factorial disorder that is associated with a tremendous financial burden, increased stress to families, and adverse academic and vocational outcomes (15). Across the life span, the social and societal costs of untreated ADHD are considerable. Many adults with ADHD display frequent changes in employment, academic underachievement, poor planning abilities, messiness, dangerous driving, and instable relationships or tend to social isolation and engagement in leisure activities that are highly absorbing or stimulating and hazardous (16). They also express difficulties in housekeeping and managing their children (17). ADHD is also a common and costly condition affecting performance at work. In a national representative sample of workers in the USA a total of 4.2% suffer from ADHD which results in 35 days of annual lost work performance (18). Finally, it is important to mention that for an ADHD diagnosis in adults functional impairment in domains of life is required and psychotherapy may be decisive in the management of the mentioned functional impairment (19).

Psychotherapeutic treatment of adult ADHD is considered as part of a comprehensive multimodal therapy program which is based on pharmacological treatment. According to NICE (12, 13) pharmacological and psychotherapeutic treatments are both possible interventions. Although the importance of psychotherapy is continuously emphasized, controlled studies to support this claim are still scarce and the effectiveness of psychotherapy in adult ADHD is partly inconsistent. Therefore, the level of evidence (III, recommendation level C) is still inconsistent. Moreover, there are no scientific criteria available to determine in which cases a combined therapy is more effective than exclusive pharmacotherapy since studies on this topic are still lacking (evidence level IV, recommendation level D). Nevertheless, the guidelines recommend to combine the two forms of therapy since some symptoms are more accessible by psychotherapy (e.g., organizational and social behavior) whereas other symptoms respond more readily to medication (e.g., attention, emotional instability) [see (12, 13)].

Multimodal treatment including behavioral therapy and medication has been found to be most effective in treating children with ADHD (16). The *Multimodal Treatment of Attention Deficit Hyperactivity Disorder* (MTA) study likewise documented that a comprehensive behavior therapy program is also effective, albeit less than medication. A multimodal program which combined medication and behavioral interventions results in the greatest overall improvement (20). According to Kordon and Kahl (21) the rationale for multidimensional therapy is again a differential response of symptoms to various treatment strategies which implies that not all symptomatic areas can be improved by a pharmacological treatment alone. For example, compliance and permanent changes in behavior can be improved by means of detailed psychoeducation for the patient and his family. Furthermore, in many patients comorbid disorders are present which must be considered adequately in therapy. Recommendations in the literature usually suggest cognitive-behavioral interventions (22). Occasionally analytically orientated approaches are mentioned often focusing on rather secondary aspects of the disorder such as the frequently present problem of low self-esteem. Recommendations in the literature favor almost exclusively the application of cognitive-behavioral interventions [e.g., (23, 24)]. Different authors [e.g., (25)] point explicitly at the fact that unstructured interventions are ineffective in ADHD patients due to frequent neuropsychological deficits caused by the disorder itself. Difficulties in executive functioning resulting in lack of focusing and problems with memory, and difficulties in the task performance ask for a structured psychotherapeutic approach.

With regard to the indication for psychotherapy specific arguments stem from studies on the course of ADHD on the one hand and from the results of pharmacological studies on the other hand. With regard to the course of ADHD Ramsay and Rostain (26) state: “rather than ‘growing out of’ ADHD, the symptoms seem to ‘grow with’ the individual” (p. 320). In the same way Biederman et al. (27) report in their study on the course of ADHD in children that at the transition to adulthood very different presentations of the disorder are distinguishable. Apart from a so-called “syndromatic remission” (<8 of the 14 possible DSM-III-R criteria) they distinguish a “symptomatic remission” (<5 symptoms, so-called subthreshold) and a “functional remission” (<5 symptoms and no functional impairments). Depending on the definition a different quantity of patients showed remarkable impairments. Especially in attention-deficits the relatively lowest improvements were observed. Moreover, it is assumed that not all changes appear simultaneously. Symptoms tend to get better within weeks, functioning gets better within months and—perhaps most important—careful observation may identify changes in development taking place over years. Therefore, psychosocial treatment is helpful to reduce any residual impairment (28).

Psychopharmacological studies put the emphasis on the convincing efficacy of methylphenidate [MPH, e.g., (29)], but they also show that in some patients efficacy is low or medication is refused (19, 30). Treatment denial may be a consequence of a lack of proper attitude or depend on a general opinion concerning medication or possibly is the result of side effects. In addition, pharmacotherapy despite of its verified efficacy is not consistently positive assessed. Lower creativity levels in everyday life or a changed perception of oneself and others are sometimes newly emerging problems (31). Responder rates of MPH in adults as reported in literature vary from 25 to 78% (32) and emphasize that a big number of patients, if not even all of them, still have at least some persisting symptoms at the end of their therapy.

Furthermore, due to the persistence of ADHD symptoms various problems in different areas of functioning arise (e.g., family and social life, work, and organization). Even in the case of an excellent therapeutic response these problems do not disappear immediately. Demoralization caused by long lasting disappointment and reoccurring frustration is frequently present and has to be considered in therapy (33). Based on clinical observations and reports from affected people some ADHD patients wish to be treated by other means than medication by receiving additional help which often results in essential and ongoing changes in everyday life (19). Self-esteem is—as already mentioned—frequently affected by the long-lasting problems caused by ADHD. Relevant targets for psychotherapy are found in the following areas which are associated with the disorder itself [e.g., (34)]: area of performance, social and interpersonal area, and problems with adaptive behavior.

Despite of the continuous general demand for multimodal treatment, the data presented are still insufficient. An important element of almost all existent studies is pharmacological treatment usually with MPH which is in accordance with today's state of knowledge. Psychotherapy on its own does not seem to be as efficacious as pharmacotherapy which is comprehensible considering the current knowledge about etiology and pathology of this disorder.

Cognitive-behavioral therapy (CBT) shows some evidence in the psychological treatment of ADHD. Different un- and controlled CBT studies reported some treatments effect like ADHD symptom reduction [e.g., (24, 35–39)]. A review and meta-analyses of nine randomized controlled trials showed that CBT for adult ADHD was superior to waiting list with a moderate to large effect size (*SMD* = 0.76, 95% *CI* [0.21, 1.31], *p* = 0.006) and superior to active control groups with a small to moderate effect size (*SMD* = 0.43, 95% *CI* [0.14, 0.71], *p* = 0.004) in reducing ADHD symptoms post-intervention (40).

In conclusion, recent empirical data about psychotherapeutic treatments of patients with ADHD indicate that psychotherapy might be efficacious in the treatment of adult ADHD. However, it is not possible at the present time to give a concluding appraisal of the efficacy of a combined pharmacological and psychotherapeutic intervention in comparison to a purely pharmacological treatment due to major deficits of studies. Trying to summarize the problems of the studies, two problem areas can be identified: problems concerning content and concepts (e.g., selection of particular therapeutic components often not justified sufficiently, often manuals are missing or too general and not enough standardized, components which are also applicable for group settings are often not much individualized), and problems concerning methodological aspects [non-randomized controlled study, diagnostic interviews not standardized (ADHD and comorbid conditions), comorbidity not recorded, pharmacotherapy not controlled or not standardized, control groups missing, control groups without controlling relevant variables (e.g., contact time with mental health services), treatment response limited to self-report instruments, rarely multimodal assessment, assessment of post-treatment outcome only, no follow-up period to investigate maintenance of outcome].

General aim of the study was to evaluate the contribution of psychotherapy to the treatment of adult ADHD patients. The primary objective of the randomized controlled study was to evaluate the efficacy of multimodal therapy (treatment group: medication + psychotherapy) compared with medication alone (control group: medication) in adult subjects with ADHD. We expected that the multimodal treatment shows a greater success in treatment than medication alone. Secondary objectives were: assessment of the stability of the change in follow-up, assessment of the benefits with regard to impairments in daily life, and assessment of the quality of the psychotherapy.

## Methods

### Subjects

A total of 50 individuals who came to the ADHD Special Consultations Unit of the Outpatient Department of the University of Basel Psychiatric Clinics (UPC) between 2010 and 2015 were recruited for this randomized controlled study. Five men and two women dropped out before being randomized to one of the two treatment groups. Finally, the data of 43 ADHD patients were analyzed.

Male and female patients with a diagnosis of ADHD according to DSM-IV criteria, with symptoms before 7 years that continue to meet these criteria at the time of assessment, were enrolled in the study. ADHD was not diagnosed if the symptoms were better accounted for by another psychiatric disorder (e.g., mood disorder). All individuals fulfilled all general criteria for a treatment with MPH in accordance with the existing guidelines (12, 13) and preconditions of psychotherapy (sufficient language skills, appropriate intellectual level corresponding to clinical impression). Comorbid psychiatric disorders were allowed, if the subject was in a stable clinical condition and/or has been on a stable dosage for at least 3 months prior to screening.

Subjects with clinically significant unstable medical or neurological conditions like hypertension and other cardiac diseases, hyperthyroidism, epilepsy, tic-disorder, or any other severe somatic disorder were excluded from the study. Also, individuals with unstable psychiatric disorders apart from ADHD like moderate to severe mood or anxiety disorders, schizophrenia, with an addiction to or abuse of cocaine, heroin, or other opioids, designer drugs, tranquilizers, other sedatives, or alcohol, and those consuming THC more than two joints a week or more than eight joints a month were also excluded. Addiction to or abuse of nicotine and/or caffeine were documented but were not exclusion criteria. Acute psychiatric symptoms like suicidality, psychotic exacerbations, manic episode, known non-response or allergy to MPH, pregnant, or breast-feeding women were also exclusion criteria. Finally, individuals who received CBT within the last 2 years could not participate in the study.

### Study design and procedure

**Figure 1:**
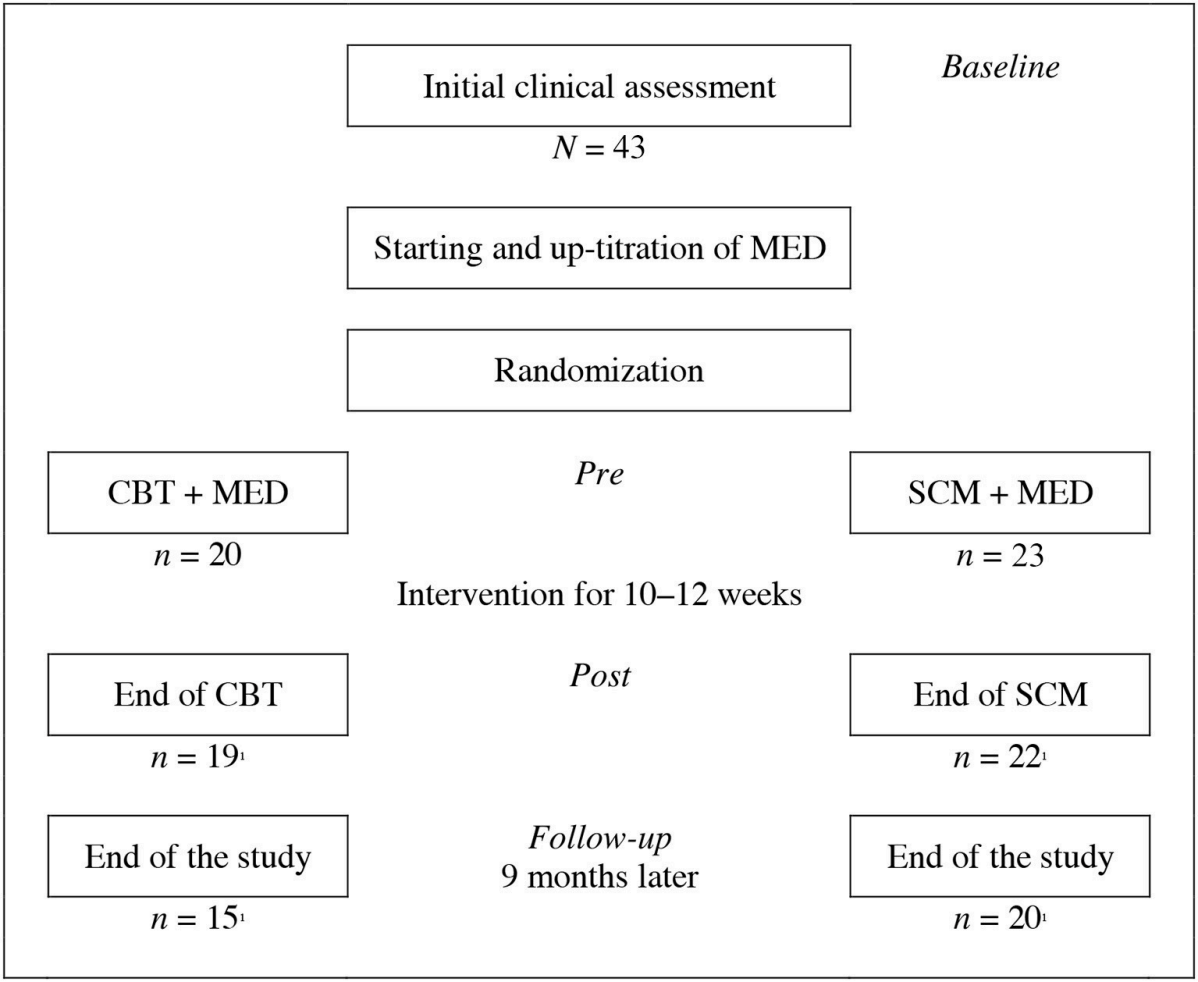
Study design and procedure (baseline, pre, post, and follow-up evaluation). MED, Medication; CBT, Cognitive-behavioral therapy; SCM, Standard clinical management; ^1^Patient(s) refused further participation.

All subjects presenting to obtain a diagnosis, underwent a comprehensive diagnostic assessment to assess the existence of the diagnosis of adult ADHD and possible comorbid disorders (see Figure 1). After confirmation of diagnosis and signature of informed consent to participate in the study, treatment was initiated with MPH since MPH has been considered to be the treatment of first choice for ADHD [see (32)], as shown in several empirical studies [e.g., (41)] as well as a meta-analysis (29). This is also stated in general reviews or guidelines [e.g., (11–13)]. Other concomitant medication was only allowed if the subject had been on a stable dosage for more than 3 months prior to screening. Up-titration of MPH was considered successful if there was a response of at least 30% in the CAARS, and/or MPH dosage was stable for at least 2 weeks. Subsequently, patients were randomly allocated to one of the two treatment groups, when the MPH medication was stabilized at their optimum dose but still show clinically significant symptoms (CAARS > 20). Allocation to treatment groups was done according to a stratified block randomization [strata: age and sex; see (42)]. As indicated by previous therapeutic studies no other variables are known to be of importance in randomization. The treatment group received in addition to MPH a psychotherapeutic treatment. It consisted of maximum 12 therapeutic sessions. The control group received clinical management that meant the same number of contacts as the psychotherapy group. However, these contacts were focused on pharmacotherapy. The patients were asked about symptoms and any possible side effects. Specific cognitive-behavioral interventions may not be performed during these contacts.

At the same time point, baseline evaluation was done. After completion of therapeutic interventions, a post-study evaluation was done. Nine months later, a follow-up assessment of outcome variables was performed to investigate the sustainability of treatment responses. All ratings were conducted by independent trained clinical psychologists who did not know which participants belong neither to the psychotherapy nor to the clinical management group.

The psychotherapeutic treatment was performed by trained psychologists with certification in clinical psychology and extended experience in CBT. The effective use of manual-based treatments must be preceded by adequate training (43). Therefore, prior to the start of the study, a 5 days' workshop was performed by the principal investigators to train the involved psychotherapists. The proper use of a manual-based therapy requires flexible application and ongoing supervision (43). Therefore, all therapists received supervision on a regular base. The supervisor examined treatment adherence and the quality of delivery by continuous supervision. Furthermore, the supervisor supported the therapists if problems rose.

The medication was done at the Outpatient Department of Psychiatry of the UPC by a psychiatrist who had experiences in treating adult ADHD.

### Assessment instruments

Because of the various impairments in ADHD patients multidimensional clinical diagnostic instruments were applied. Apart from the confirmation of the diagnosis and the recognition of possible comorbid diagnosis, we aimed to use a multimodal approach (44). We had to consider different sources of data (e.g., patient, independent rater) and different functional ranges (e.g., experiences, behavior, feelings, working capacity). Details about the instruments are described below.

#### Classification

In order to verify the diagnosis and to diagnose comorbid disorders internationally well-established diagnostic interviews were applied:

##### Structured clinical interview for DSM-IV [SCID-I and SCID-II; German version (45, 46)]

Structured interview to assess disorders according to DSM-IV Axis-I (e.g., affective disorders) und Axis-II disorders (personality disorders); used to assess comorbid disorders, administered only at pre-treatment.

##### Conners' adult ADHD diagnostic interview for DSM-IV [CAADID; (47)]

Structured interview based on the DSM-IV criteria and used for assessing diagnosis retrospectively or confirming a currently obvious disorder.

#### ADHD symptomatology

With respect to the determination of the pathology there is no generally accepted instrument until now which could be considered as a gold standard (48). Therefore, those instruments have been selected which were applied in studies before, in order to assure comparability, reliability, and validity.

##### Conners' adult ADHD rating scale [CAARS; (49)]

Eighteen items rating scale according to the DSM-IV criteria; four-point rating scale (severity or frequency of the symptoms), clinician administered.

##### Conners' ADHD adult rating scale self-report short version [CAARS-S:S; (49)]

Twenty-six items rating scale based on DSM-IV criteria.

##### Conners' adult ADHD rating scale observer long version [CAARS-O:L; (49)]

Sixty-six items; four-point rating scale (severity or frequency of the symptoms), scoring: inattention/memory problems, hyperactivity/restlessness, impulsivity/emotional lability, problems with self-concept, DSM-IV inattentive symptoms, DSM-IV hyperactive symptoms, DSM-IV ADHD symptoms total, ADHD index.

##### Wender-reimherr adult attention deficit disorder rating scale [WRAADDS; German version (50)]

Structured interview according to the criteria of Paul H. Wender; seven syndromes (inattention, hyperactivity/restlessness, impulsivity, disorganization, affective lability, stress intolerance, temper), more adequate to assess the symptomatology in adults.

##### ADHD self-rating behavior questionnaire [ADHD-SR; (51)]

Twenty-two items self-rating scale; 18 items according to DSM-IV/ICD-10; 4 items to assess aspects like impairment, beginning of symptomatology.

#### Impairment and quality of life

ADHD symptomatology is always related to more or less remarkable impairments in different functional areas of life. Despite significant impairments related to adult ADHD, little is known about how ADHD symptomatology and functional impairments impact quality of life in patients with this disorder [see (52, 53)]. Especially in the case of chronically progressing disorders, such as for instance ADHD, this is of great significance and has to be assessed. The following questionnaires were applied:

##### Sheehan's disability scale [SDS; (54)]

Global self-rating scale to assess in work, social life or leisure activities and home-life or family responsibilities.

##### Adult attention-deficit/hyperactivity disorder quality-of-life scale [AAQol; (52)]

Twenty-nine items self-rating scale; four domains: life productivity, psychological health, relationships, life outlook.

##### General perceived self-efficacy scale [SWE; (55)], SWE

The SWE is a unidimensional scale containing 10 items which are answered on a four-point scale.

##### Rosenberg self-esteem scale [RSES; (56)]

The RSES is a unidimensional scale containing 10 items which measure “global self-esteem.” Each of the 10 items is answered on a four-point scale.

#### Attitudes to treatment and quality of therapeutic process

##### Psychotherapy motivation questionnaire [fragebogen zur psychotherapiemotivation (FMP)]

The questionnaire by Schneider et al. (57) was applied to evaluate therapeutic attitude. The FMP covers several dimensions: experience of illness, explanation of illness, treatment expectation, and attitude to psychotherapy.

##### Bern post session report (58)

The questionnaire addresses central aspects of the therapeutic process. The scale, containing 29 items, is filled out by the patient, respectively, the therapist. The following areas are covered: progress within the therapy sessions, progress outside the therapy sessions, quality of therapy relation, therapy satisfaction, and emotion in the therapy. The function of this questionnaire is to register the progression of the therapy and eventually to provide more details about the interpretation of the results found in this study.

### Medication

All subjects received a standard treatment with MPH which was administered as follows: initiation of treatment with 20 mg Ritalin® LA 20 mg/day; up-titration of Ritalin® every fourth day up to a level of 0.5–1.3 mg/kg bodyweight/day; stabilization of final dosage for at least 2 weeks and no increase of dosage from then of more than 10% of the final dosage. If Ritalin® LA did not show sufficient clinical effects or was not tolerated Concerta® was used as a second choice. The initial dosage was Concerta® 18 mg/day, followed by an up-titration as outlined above.

No other stimulants apart from long-acting MPH were used in the study. No non-stimulant medication was initiated. Subjects on non-stimulant medication with a known effect on ADHD symptoms could participate in the study if they fulfilled the following conditions: stable dosage for the last 3 months previous to baseline investigation, sufficient remaining symptoms for ADHD diagnosis. In these subjects MPH was administered in addition to the existing medication. Subjects on non-stimulant medication for other medical or psychiatric conditions could participate if their medication was administered on a stable dosage for at least 3 months and the underlying disorder is remitted to a clinically not significant level of symptoms. In addition, this particular medication must not interfere with MPH.

As soon as subjects were on a clinically sufficient dosage of MPH and on a stable level for at least 2 weeks, they were randomized to either multimodal therapy or medication alone. At the end of the study there was a short clinical check-up again and adequacy of medication was controlled again. Afterwards subjects were referred to their physicians or to a specialist for ADHD if they wished to continue treatment.

The main outcome measure concerning initiation and dosage adjustment of MPH was a reduction of 30% or more in the sum score of the investigator-rated CAARS. As soon as patients reach this level of individual reduction in sum scores their dosage was not increased any further. If subjects remained stable on this dosage for at least 2 weeks they were randomized to one of the two treatment groups in the study.

### Psychotherapeutic treatment program

The psychotherapeutic program was a cognitive-behavioral program designed for single-settings and contained several modules which were summarized in a standardized manual (see Table 1). The selection and composition of the modules were directed by the following criteria [see (59)]: ADHD pathology in adulthood, as outlined by Wender (60) or Wilens and Dodson (16); already published therapeutic approaches (61, 62); general principles of CBT; analysis of problem areas of more than 400 own patients as well as our own clinical experience in the treatment of ADHD patients.

**Table 1:** Overview of the psychotherapeutic modules.

**Psychotherapeutic modules**		**Number of sessions (each 120 min)**	**Contents and themes**
**BASIC MODULES (B)**			
B 1	Psychoeducation and behavior analysis	2	• Psychoeducation about ADHD in adulthood
			• Information about vulnerability factors relative to ADHD pathology
			• Analysis of the specific behavioral problems
			• Looking for strategies that have been successful up to now
B 2	Cognitive therapy	4	• Therapy model and introduction to cognitive therapy
			• Restructuring dysfunctional ADHD-specific cognitions and fundamental beliefs or rather cognitive schemas, cognitive distortions and errors
B 3	Final and preventive session	1	• Summary of helpful strategies
			• Placing emphasis on the necessity of doing exercises continuously
			• Strategies supporting the self-evaluation of further improvements
**OPTIONAL MODULES (O)**			
O 1	Organization	1	• Acquisition of organizing and planning skills
O 2	Procrastination	1	• Handling procrastination of duties and activities
O 3	Learning	1	• Acquisition of learning skills
			• Training concentration
O 4	Impulsiveness	1	• Handling impulsiveness, emotional overreactivity and low control of affect
O 5	Partnership	1	• Improvement of communication and problem-solving skills in close relationships

The full therapy program consisted of a minimum of 10 weekly sessions (including one final session), and a maximum of 12 sessions. The manual contained a description of each session of the therapeutic interventions as well as general advices. (e.g., example of engagement with the patients). Apart from the first two sessions the weekly therapy sessions were identical in structure: discussion of homework given in the previous session, presenting the theme of the current session, presentation of new homework.

Discussing homework of the previous session aimed to evaluate therapeutic progress and to control whether therapeutic strategies were used in daily life or not. If problems raised it was necessary to identify the cause and to work out realistic solutions with the patient. The presentation of the content of each session should provide a clear structure for the patient. This seems to be extremely important in the treatment of ADHD (23, 26). The most important part of each session was the main topic of the module in question. Homework was always derived from this main topic. Patients were asked to do their homework and write down their experience with the tasks they had to fulfill.

Practical parts of the therapy sessions were: exercises, role plays, hand-outs, and work sheets. The therapy set consisted of different basic (B 1–3) and optional modules (O 1–5). Whereas, the basic modules were used with all patients, the application of the optional modules depended on the conclusions from the individual analysis of behavior which meant that the specific problems of the patient were taken into account for the choice of optional modules.

### Standard clinical management

Concerning standard clinical management (SCM) we refer to the NIMH-depression study (63). In the NIMH-study a 30-min medication counseling on effects and side effects was administered to patients of the control group by a psychiatrist. The frequency was the same as in the experimental group. Elkin (64) described it as “minimal supportive therapy” which is more than a waiting group.

In our study patients in the control group received SCM consisting of medication counseling administered by a psychiatrist. SCM was administered weekly by 30 min sessions. The elements of SCM were written down in a manual to guide the psychiatrist through the standardized procedure similar as described by Fawcett et al. (65).

In SCM apart from evaluating the general state of health and mind as well as effects and side effects of medication, the pulse rate, blood pressure and weight were measured. Also the CAARS was administered to assess the course of ADHD with ongoing medication. The patient was encouraged to report difficulties he experienced since the last visit but he was instructed that he will not receive any psychotherapeutic interventions. The psychiatrist listened and responded empathically but restricted counseling to the above-mentioned items. In the case of severely aggravating difficulties or even suicidality the psychiatrist was obliged to react to the patient's problems in the same way as if the patient was not a study participant. If additional psychotherapeutic counseling, additional medication, or even hospitalization became necessary the patient was withdrawn from the study and received the usual clinical treatment. This procedure ascertained that patient's needs were fully respected and dangerous situations were avoided. Psychiatrists received a didactic training in SCM for at least half a day.

### Data analysis

For all statistical analyses, SPSS (Version 24.0) were used. In a first step, descriptive statistics were determined, and the sample was explored for possible group differences at the baseline. To accurately answer the main hypothesis that a treatment of ADHD with CBT plus medication (MED) would provide a higher efficacy than SCM plus MED, in a second step analyses of variance (ANOVA) with the between-factor (*group*: CBT + MED vs. SCM + MED) and the within-factor (*time*: pre, post, follow-up), and—for the main hypothesis most relevant—the interaction term (*group* × *time*) were conducted. To provide robust outcomes, normalized latent variables were extracted through principal component analysis for the total scores and the different domains of ADHD assessed via CAARS, CAARS-S:S, CAARS-O:L, WRAADDS, and ADHD-SR and for impairments measured by SDS, AAQol, SWE, and RSES. Since this data set does not contain a suitable number of cases for such a procedure to produce stable results, the general data set of the ADHD Special Consultations Unit of the Outpatient Department of the UPC was used to provide the weights of the different subscales. For further information on this data set see [(5), (7) or (66)]. Finally, to gain broader insight in the therapy efficacy, the relations of CBT process characteristics within this group and the symptom and impairment improvements were examined using Spearman's correlations.

## Results

### Participants characteristics

The sample of the study consisted in 24 (55.8%) males and 19 (44.2%) females. The subjects were between 18 and 49 years of age (*M* = 31.91, *SD* = 8.41). Finally, all included patients (*N* = 43) were randomized into the following two groups: Group 1: CBT + MED (*n* = 20, 46.5%); and Group 2: SCM + MED (*n* = 23, 53.5%). The distribution of the sociodemographic information and the current clinical characteristics, the symptom and impairment levels of these groups at time of the first patient's contact are shown in Table 2. No significant group differences at baseline were found.

**Table 2:** Sociodemographic and current clinical characteristics of the patients in the two treatment groups.

	**CBT + MED *n* = 20**	**SCM + MED *n* = 23**	**Test values**	***p***
Gender			$\chi_{\left(1\right)}^{2}$ = 0.01	0.920
Male	11	13		
Female	9	10		
Age [*M* (*SD*)]	34.05 (9.34)	30.04 (7.21)	*F*_(1)_ = 2.52	0.120
Relational status			$\chi_{\left(3\right)}^{2}$ = 2.80	0.424
Single	10	13		
Married/relationship	9	6		
Divorced	1	3		
Separated	0	1		
Education			$\chi_{\left(3\right)}^{2}$ = 1.36	0.714
Secondary School	11	13		
College	7	8		
University	2	2		
Employment			$\chi_{\left(5\right)}^{2}$ = 4.12	0.532
Fulltime	5	4		
Part-time	4	3		
Unemployed	2	2		
Student	8	9		
Homemaker	0	4		
Unemployable	1	1		
ADHD				
*Symptom levels (latent factors)* [*M* (*SD*)]				
Total	1.32 (0.25)	1.30 (0.27)	*t*_(40)_ = 0.335	0.739
INA	1.41 (0.23)	1.34 (0.24)	*t*_(40)_ = −0.941	0.353
HYP	1.05 (0.31)	1.06 (0.43)	*t*_(40)_ = 0.099	0.922
IMP	1.01 (0.38)	1.03 (0.30)	*t*_(40)_ = 0.125	0.901
ES	1.08 (0.25)	1.18 (0.42)	*t*_(34.44)_ = 0.920	0.364
*Impairments (latent factors)* [*M* (*SD*)]				
IMPAIR 1	2.01 (0.48)	1.97 (0.53)	*t*_(38)_ = −0.722	0.477
IMPAIR 2	−0.14 (0.35)	−0.07 (0.40)	*t*_(38)_ = −0.543	0.592
Comorbid disorders				
*Axis I*	2	6	$\chi_{\left(1\right)}^{2}$ = 0.00	0.975
Alcohol abuse	0	1		
Cannabis	0/1	1/0		
abuse/dependence				
Depressive episode	1	1		
Social phobia	0	1		
Specific phobia	0	1		
PTSD	0	1		
*Axis II*	6	2	$\chi_{\left(5\right)}^{2}$ = 6.56	0.256
Avoidant PD	1	2		
Obsessive-compulsive PD	1	0		
Paranoid PD	1	0		
Borderline PD	1	0		
Antisocial PD	2	0		
Attitude to treatment (*n* = 19) [*M* (*SD*)]	164.32 (12.74)^1^	–	–	–
Experience of illness	29.74 (4.20)^2^	–		
Explanation of illness	28.00 (4.86)^3^	–		
Treatment expectation	31.58 (3.02)^4^	–		
Attitude to psychotherapy	75.00 (7.34)^5^	–		

CBT, Cognitive-behavioral therapy; SCM, Standard clinical management; MED, Medication; M, mean; SD, standard deviation; ADHD, Attention-deficit/hyperactivity disorder; INA, Inattention; HYP, Hyperactivity; IMP, Impulsivity; ES, Emotional symptoms; IMPAIR 1/2, Impairment factor 1/2; PTSD, Post-traumatic stress disorder; PD, Personality disorder;

1 t-value, 51; percentile, 54;

2 t-value, 45; percentile, 31;

3 t-value, 49; percentile, 48;

4 t-value, 55; percentile, 69;

5 *t-value, 53; percentile, 62*.

### Extracting latent variables for ADHD symptoms and impairment

To provide robust outcomes measures, firstly latent variables were formed. For the general ADHD symptoms a factor analysis was executed; to include only the shared variances of the different perspectives within the different ADHD scales the principle axis method was used. A solution with one component was found (λ = 3.21), explaining 64.20% of the total variance. The same procedure was used for each subdomain where also solutions with one component were repeatedly found (λ = 1.85–3.51), explaining 56.36% of the variance in the subscales of inattention, 70.19% of hyperactivity, 61.71% of impulsivity, and 61.59% of emotional symptoms. For the impairment scales a solution with two factors was found (λ_i1_ = 3.98 and λ _i2_ = 1.65), explaining 56.25% of the variance. On the first rotated factor all subscales of AAQoL (0.566–0.681) and the subscales of SDS for family (0.542) and work (0.398) loaded highly; while the subscale of SDS for leisure (0.318) and social contacts (0.319) loaded on both factors medium high. Subscales of RSES and SWE loaded both highly on the second factor (0.641–0.862). Those two factors correlated slightly (*r* = 0.362).

### Symptom level changes and impairment changes between the two treatment groups

**Figure 2:**
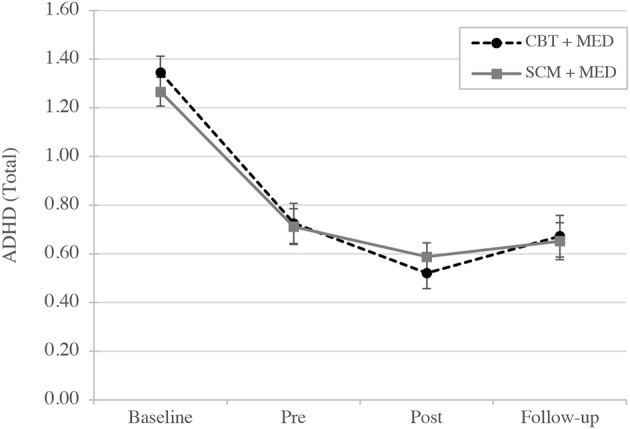
Mean of latent variables: ADHD symptoms changes over time (baseline, pre, post, and follow-up evaluation). CBT, Cognitive-behavioral therapy; SCM, Standard clinical management; MED, Medication.

**Figure 3:**
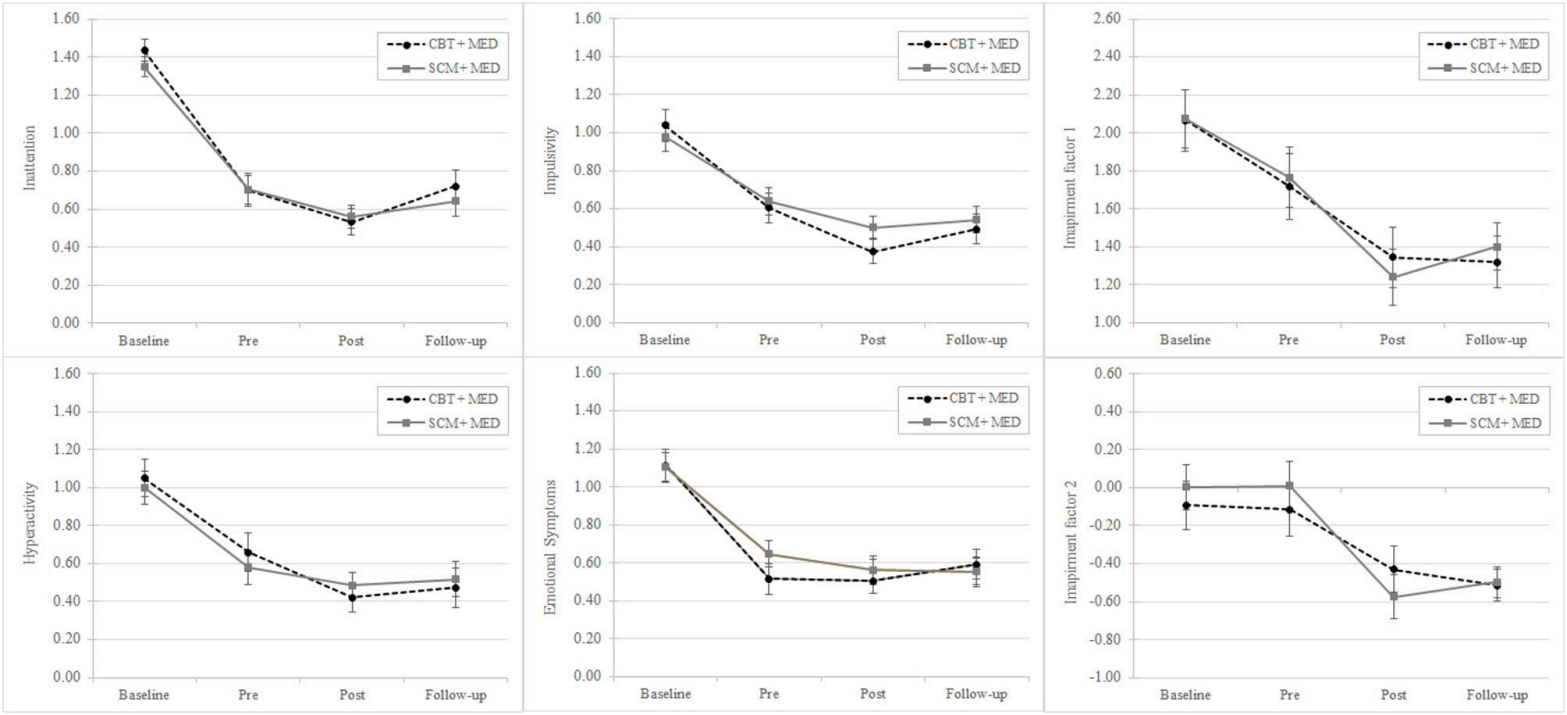
Mean of latent variables: ADHD symptoms (inattention, hyperactivity, impulsivity, and emotional symptoms) and impairments changes over time (baseline, pre, post, and follow-up evaluation). CBT, Cognitive-behavioral therapy; SCM, Standard clinical management; MED, Medication.

To test the main hypothesis ANOVAs between subjects with CBT + MED and SCM + MED were conducted with the within factor *time*. Table 3 presents the estimated means of the two different groups. The analysis of ADHD symptoms showed a statistically significant main effect of the factor *time* [*F*_(1, 96)_ = 83.49, *p* < 0.001, η^2^ = 0.723] but not of the factor *group* [*F*_(1, 32)_ = 0.01, *n.s*.]. Neither did the interaction effect of group and time reach significance [*F*_(1, 96)_ = 0.67, *n.s*.]. The same pattern was also found for the subdomains of ADHD: inattention, hyperactivity, impulsivity, and emotional symptoms and also for the two latent variables of impairment (see Table 4, Figure 2, and Figure 3).

**Table 3:** Mean (M) and standard error (SE) of ADHD symptoms and impairment at baseline pre, post, and follow-up evaluation.

	**CBT** + **MED**				**SCM** + **MED**			
	**Baseline (*M* [*SE*])**	**Pre (*M* [*SE*])**	**Post (*M* [*SE*])**	**Follow-up (*M* [*SE*])**	**Baseline (*M* [*SE*])**	**Pre (*M* [*SE*])**	**Post (*M* [*SE*])**	**Follow-up [*M* (*SE*)]**
ADHD (Total)	1.35 (0.07)	0.73 (0.08)	0.52 (0.06)	0.67 (0.09)	1.27 (0.06)	0.71 (0.07)	0.59 (0.06)	0.65 (0.08)
INA	1.44 (0.06)	0.70 (0.09)	0.53 (0.07)	0.72 (0.09)	1.35 (0.05)	0.70 (0.08)	0.56 (0.06)	0.64 (0.08)
HYP	1.05 (0.10)	0.66 (0.10)	0.42 (0.08)	0.47 (0.11)	1.00 (0.09)	0.58 (0.09)	0.49 (0.07)	0.52 (0.09)
IMP	1.04 (0.08)	0.60 (0.07)	0.37 (0.07)	0.49 (0.08)	0.98 (0.08)	0.64 (0.07)	0.50 (0.06)	0.54 (0.07)
ES	1.11 (0.09)	0.52 (0.08)	0.51 (0.09)	0.59 (0.09)	1.10 (0.08)	0.65 (0.07)	0.56 (0.08)	0.55 (0.08)
IMPAIR 1	2.07 (0.16)	1.72 (0.17)	1.35 (0.16)	1.32 (0.13)	2.07 (0.15)	1.77 (0.16)	1.24 (0.15)	1.40 (0.12)
IMPAIR 2	−0.09 (0.13)	−0.12 (0.14)	−0.43 (0.12)	−0.51 (0.09)	0.00 (0.12)	0.01 (0.13)	−0.57 (0.11)	−0.50 (0.08)

*Latent factors of the different scales for: ADHD, Attention-deficit/hyperactivity disorder; INA, Inattention; HYP, Hyperactivity; IMP, Impulsivity; ES, Emotional symptoms; IMPAIR 1/2, Impairment factor 1/2; CBT, Cognitive-behavioral therapy; SCM, Standard clinical management; MED, Medication*.

**Table 4:** ANOVAs of ADHD symptom and impairment level changes between the two treatment groups.

	**Main effects of** ***time***		**Main effects of** ***group***		**Interactions** ***time*** × **group**	
	***F-*value**	**η^2^**	***F-*value**	**η^2^**	***F-*value**	**η^2^**
ADHD (Total)	*F*_(3, 96)_ = 83.49^***^	0.723	*F*_(1, 32)_ = 0.00	0.001	*F*_(3, 96)_ = 0.67	0.021
INA	*F*_(3, 96)_ = 91.62^***^	0.741	*F*_(1, 32)_ = 0.20	0.006	*F*_(3, 96)_ = 0.51	0.016
HYP	*F*_(3, 96)_ = 37.87^***^	0.542	*F*_(1, 32)_ = 0.00	0.000	*F*_(3, 96)_ = 0.69	0.021
IMP	*F*_(3, 96)_ = 60.90^***^	0.656	*F*_(1, 32)_ = 0.18	0.006	*F*_(3, 96)_ = 1.39	0.042
ES	*F*_(3, 96)_ = 45.64^***^	0.588	*F*_(1, 32)_ = 0.04	0.005	*F*_(3, 96)_ = 0.88	0.027
IMPAIR 1	*F*_(3, 72)_ = 14.16^***^	0.371	*F*_(1, 24)_ = 0.00	0.000	*F*_(3, 72)_ = 0.18	0.007
IMPAIR 2	*F*_(3, 72)_ = 10.44^***^	0.303	*F*_(1, 24)_ = 0.01	0.003	*F*_(3, 72)_ = 0.56	0.023

Latent factors of the different scales for: ADHD, Attention-deficit/hyperactivity disorder; INA, Inattention; HYP, Hyperactivity; IMP, Impulsivity; ES, Emotional symptoms; IMPAIR 1/2, Impairment factor 1/2;

*** *p < 0.001*.

### Correlations between changes in core and emotional symptoms of ADHD, impairments and process characteristics of the CBT

Spearman's correlations were carried out to explore the relationship between CBT process characteristics of the therapy group (progress within therapy) and the changes in symptom and impairment levels. Emotion in therapy showed for the symptoms inattention (ρ = −0.57, *p* < 0.01), hyperactivity (ρ = −0.60, *p* < 0.01), and emotional symptoms (ρ = −0.60, *p* < 0.01) high correlations but not for impulsivity (ρ = −0.28, *n.s*.). The perceived quality of therapy relation and therapy satisfaction correlated significantly with the latent factors of impairment (factor 1: ρ = −0.46, *p* < 0.01 and ρ = −0.56, *p* < 0.01; factor 2 ρ = −0.49, *p* < 0.01 and ρ = −0.57, *p* < 0.01) but not with ADHD symptoms. All correlations are displayed in Table 5.

**Table 5:** Spearman's correlations between changes in core and emotional symptoms of ADHD, impairments and process characteristics of the CBT.

	**INA**	**HYP**	**IMP**	**ES**	**IMPAIR 1**	**IMPAIR 2**
Progress within therapy	−0.343	−0.353	−0.225	−0.142	−0.386	−0.460^*^
Progress outside therapy	−0.379	−0.278	−0.265	−0.204	−0.374	−0.415
Quality of therapy relation	−0.309	−0.363	−0.198	−0.137	−0.464^*^	−0.494^*^
Therapy satisfaction	−0.293	−0.408	−0.242	−0.155	−0.560^**^	−0.568^**^
Emotion in therapy	−0.568^**^	−0.603^**^	−0.279	−0.613^**^	−0.400^*^	−0.412^*^

Difference of post to baseline latent factors INA, Inattention; HYP, Hyperactivity; IMP, Impulsivity; ES, Emotional symptoms; IMPAIR 1/2, Impairment factor 1/2;

* p < 0.05;

** *p < 0.01 (one-tailed)*.

## Discussion

The aim of the randomized controlled study was to analyze the efficacy of multimodal individual therapy (experimental group) in comparison with medication alone (control group). Whereas, the experimental group (CBT + MED) attended individual psychotherapy sessions, the control group (SCM + MED) only received medication and clinical counseling. The current findings do not support the notion that CBT outperforms SCM when added to a pharmacological treatment.

The different analyses show a statistically decrease of ADHD symptoms during the time (baseline, pre- and post-treatment) and an increase of symptoms in the follow-up. However, both groups did not differ significantly in the ratings of ADHD symptoms. The same results were found for the single ADHD domains (inattention, hyperactivity, impulsivity, and emotional symptoms) and also for impairment in daily life. The assessment of the quality of therapy in the therapy group showed that inattention, hyperactivity, and emotional symptoms correlated high and significantly with the domain emotion in therapy. However, the perceived quality of therapy relation and therapy satisfaction correlated significantly with impairments but not with ADHD symptoms.

According to guidelines, psychopharmacological treatment is currently considered as first-line treatment for adults in ADHD (12, 13, 19, 67). However, the course of the disorder seems to justify additional psychological interventions for several reasons: Some symptoms seem to be more accessible by psychotherapy (e.g., organizational and social behavior) whereas other symptoms respond more readily to medication (e.g., attention, emotional instability) (11). In addition, the full remission of ADHD symptoms is not always achieved with pharmacotherapy (68). Some of the specific impairments, viewed as consequences of the chronic course of ADHD from early childhood into the adult life span, resist to pharmacology, and demand a treatment option. Finally, either due to skepticism toward medication or due to lack of tolerance of psychopharmacological interventions (19, 69), it seems to be evident that psychotherapy could help ADHD patients to treat particularly issues in cognitive schemas, organization, procrastination, learning, emotional regulation, self-esteem, and/or partnership.

A multimodal approach to the treatment of ADHD is recommended by international guidelines (12, 13). CBT may be a supplement to psychopharmacological treatments, but more research is needed to establish the effectiveness of this treatment. The findings of the small number of randomized controlled studies in this area and particularly in individual psychotherapy for adults with ADHD have reported mixed findings.

In line with the findings of the current study are the results of Philipsen et al. (70) who however amongst others evaluated the efficacy of CBT group + MED compared with individual clinical management + MED. The outcomes showed that the CBT group was not more effective than the individual clinical management. Two studies similar to the present study compared CBT + MED vs. MED alone (35, 62) contrasting with the results of the here presented trial. In Safren et al. (62) and Emilsson et al. (35) CBT + MED was more effective than MED alone reducing the core ADHD symptoms significantly in fact in clinician and self-rating. In addition, Emilsson et al. (35) found in the multimodal group a significant reduction of self-reported inattention, but no significant effect in self-reported hyperactivity-impulsivity.

An explanation for our results may be that in our sample the medication responded very good. All patients received Ritalin® LA. The second choice of medication (Concerta®) was not used because all patients responded very good to Ritalin® LA. The reduction of ADHD symptoms from baseline to pre-treatment assessment counted for both groups 50%. In comparison Stevenson et al. (38) reported a reduction of ADHD symptoms of 37% (baseline to pre-treatment) and at post-treatment the statistically significant reduction was 55%. Safren et al. (24) included patients with all sort of ADHD medication and in contrast to our study medication discontinuation did not lead to a dropout. Also Solanto et al. (71) reported the change of medication in their trial. No study measured and controlled the response of medication, arising the question if patients with a low response to pharmacological treatment benefit more from CBT. In general, more attention to medication should be paid in further studies, to understand in detail the benefit of medication reducing ADHD symptoms and in combination to CBT (17, 40). Studies with an additional group—CBT without medication—could help to analyze better the role of medication as well as of CBT.

In our study patients in the control group received SCM which elements were standardized and noted in a manual to guide the psychiatrist performing practice care in a professional, emphatical, and optimal way. As a consequence, SCM as an established and well-known procedure in psychiatric settings, in combination with medication, showed similar outcomes than the here presented standardized ADHD psychotherapy program. This program may not have been sufficiently effective to outperform SCM.

Whereas, every single psychotherapeutic session was video recorded and supervised, the SCM sessions unfortunately were neither recorded nor supervised. The adherence to the SCM protocol was so not controlled. However, these data could help to understand the interaction between medication, psychotherapy, and attitude to treatment in the different groups. In a smaller sample of the psychotherapy group we analyzed 27 randomly selected psychotherapy sessions of nine patients with the aim to evaluate the treatment adherence of the involved psychotherapists: Two trained independent psychologists rated on the basis of the *Cognitive Therapy Adherence and Competence Scale* [CTACS; (72)] the video recordings. The adherences of the three involved psychotherapists to the CBT program (*M* = 5.82 [*SD* = 0.004]–*M* = 5.66 [*SD* = 0.19]) and quality of therapy (*M* = 5.52 [*SD* = 0.22]–*M* = 5.72 [*SD* = 0.06]) were all above the cutoff of the CTACS (*M* ≥ 4) which can be interpreted as a good adherence.

Our ADHD psychotherapy program was based on CBT theory and models. Although the elements of our program derive from established and reliable techniques which have been adapted to the special need of ADHD patients, a pilot study may have identified less effective therapeutic interventions giving us the possibility to adjust our program. The therapy set consisted of different basic and optional modules. This set of modules was based on the diagnostic criteria of ADHD and the corresponding results from the literature (60–62). Whereas, the basic modules were used with all patients, the application of the optional modules depended on the conclusions from the individual analysis of behavior which meant that the specific problems of the patient were taken into account for the choice of optional modules. However, this procedure signifies that not all patients in the CBT + MED group passed through the same modules making it now difficult to filter the psychotherapeutic elements that are not enough effective to outperform SCM. Future research could aim to understand which components of CBT are effective in the treatment of ADHD (40).

The standardized ADHD specific CBT program consisted in 10–12 sessions, each lasting 120 min. While the treatment period might be too short to ensure that patients continued to use the new learned skills, the length of each session might be too long. Due to the compact and short treatment patients might be not able to properly develop new functional strategies, enduring behavior, and thinking patterns.

A further explanation for our results may be found in the problem activation (73) and self-awareness (74) taken place in psychotherapies. Although the aim of the psychotherapy in this study was also to activate the resources of every patients, it is important to note that only the patients of the CBT + MED group were confronted with their problems, weakness, and impairments. Lambert (75) explains that up to 5–10% of patient's symptoms during treatment increase and the length of the psychotherapeutic intervention in our study may be too short to reduce this increase of symptoms. Problem activation can trigger all kinds of protective and resistive mechanisms preventing change (73). Self-awareness may be accompanied by increased distress or lower self-esteem (74).

The current study focused on the symptom reduction of ADHD in adults. The structure of the CBT program was a central aspect in the standardized manual. The assessment of the quality of therapy in the therapy group showed that the factor emotion in therapy correlated with ADHD symptoms (except impulsivity). It seems to be beneficial to activate emotion in the therapy session. Furthermore, and more relevant, may be the findings that the quality of relation and therapy satisfaction correlated with impairments. The focus of impairments is crucial in psychotherapy with ADHD patients because of the severe difficulties of those patients on daily functioning (9, 10). ADHD symptoms increase the risk of functional impairments making it important to address this in psychotherapy. The quality of relation between psychotherapist and patient (76) is an important effect of the psychotherapy. Meta-analyses could show that 10% of the variance of the psychotherapy's success is explained of the therapeutic relationship and seems to be the most important component in psychotherapy (77). Grawe (76) stated that interpersonal relationships are important patient's resources in psychotherapy because low self-esteem and self-efficacy can be affected positively by relationships and consequently influence the process of therapy (78). Thus, future ADHD psychotherapy studies should consider more these issues.

### Limitations

The aim of our study was to analyze the data of more patients. Unfortunately, it was not possible to include more patients during the defined study time. The small size of both treatment conditions was especially relevant in the questionnaires measuring impairments. The number of filled questionnaires especially in the follow-up evaluation were too small. The package of questionnaires may be too large and must be adjusted for future studies.

Attitude to treatment was only measured and controlled in the CBT + MED group and also only with one questionnaire. However, this aspect is important in psychotherapeutic as well as in psychopharmacological treatments (75) and should be addressed in future studies. It remains unclear in our study if the patients of the SCM + MED group were poor or more motivated or had positive beliefs regarding medication. A high attitude to treatment could result in better treatment outcomes and so being able to outweigh the efficacy of psychotherapy.

Although the two groups did not show a statistically significant difference in comorbid disorders (Axis I and II), there were more patients with a comorbid personality disorder (Axis II) in the CBT + MED group. The presence of these comorbidities might have had a stronger impact on the treatment progress and effects of the experimental group. In future research the control of this aspect is mandatory.

## Conclusion

The present randomized controlled trial is one of the few studies focusing on ADHD multimodal individual therapy. The here developed standardized ADHD specific CBT program does not suggest that CBT augments significantly the reduction of ADHD symptoms. The CBT program was not able to outperform SCM which is a well-known psychiatric procedure. In further study the role of medication, CBT components, techniques, as well as modules, attitude/adherence to treatment in ADHD psychotherapy and further possible side effects in CBT should be paid more attention. This could help to understand the impact of CBT to reduce ADHD symptoms and functional impairments.

## Ethics statement

This study was carried out in accordance with the recommendations of Ethics Committee of Basel (Ethikkommission Nordwest-und Zentralschweiz) with written informed consent from all subjects. All subjects gave written informed consent in accordance with the Declaration of Helsinki. The protocol was approved by the Ethics Committee of Basel (Ethikkommission Nordwest-und Zentralschweiz).

## Author contributions

All authors listed have made a substantial, direct and intellectual contribution to the work, and approved it for publication.

### Conflict of interest statement

The authors declare that the research was conducted in the absence of any commercial or financial relationships that could be construed as a potential conflict of interest.
